# Inhibitory Control Deficits in Children with Tic Disorders Revealed by Object-Hit-and-Avoid Task

**DOI:** 10.1155/2021/8825091

**Published:** 2021-07-01

**Authors:** Nicholas Cothros, Alex Medina, Davide Martino, Sean P. Dukelow, Rachel L. Hawe, Adam Kirton, Christos Ganos, Elaheh Nosratmirshekarlou, Tamara Pringsheim

**Affiliations:** ^1^Department of Clinical Neurosciences, University of Calgary, Calgary, Alberta, Canada; ^2^Department of Clinical Neurosciences & Hotchkiss Brain Institute, University of Calgary, Calgary, Alberta, Canada; ^3^Department of Paediatrics, University of Calgary, Calgary, Alberta, Canada; ^4^Department of Neurology, Charité University Hospital Berlin, Berlin, Germany; ^5^Department of Clinical Neurosciences, Psychiatry, Pediatrics and Community Health Sciences, University of Calgary, Calgary, Alberta, Canada

## Abstract

**Background:**

Tic disorders may reflect impaired inhibitory control. This has been evaluated using different behavioural tasks, yielding mixed results. Our objective was to test inhibitory control in children with tics through simultaneous presentation of multiple, mobile stimuli.

**Methods:**

Sixty-four children with tics (mean age 12.4 years; 7.5-18.5) were evaluated using a validated robotic bimanual exoskeleton protocol (Kinarm) in an object-hit-and-avoid task, in which target and distractor objects moved across a screen and participants aimed to hit only the targets while avoiding distractors. Performance was compared to 146 typically developing controls (mean age 13 years; 6.1-19.9). The primary outcome was the percentage of distractors struck.

**Results:**

ANCOVA (age as covariate) showed participants struck significantly more distractors (participants without comorbid ADHD, 22.71% [SE 1.47]; participants with comorbid ADHD, 23.56% [1.47]; and controls, 15.59% [0.68]). Participants with comorbid ADHD struck significantly fewer targets (119.74 [2.77]) than controls, but no difference was found between participants without comorbid ADHD (122.66 [2.77]) and controls (127.00 [1.28]). Participants and controls did not differ significantly in movement speed and movement area. Just over 20% of participants with tics fell below the age-predicted norm in striking distractors, whereas fewer than 10% fell outside age-predicted norms in other task parameters.

**Conclusions:**

In children with tics (without comorbid ADHD), acting upon both targets and distractors suggests reduced ability to suppress responses to potential triggers for action. This may be related to increased sensorimotor noise or abnormal sensory gating.

## 1. Introduction

Tics are brief, intermittent, and repetitive movements or sounds, most commonly encountered in primary tic disorders [1]. Tics are suggested to result from sensorimotor disinhibition within the cortico-striato-thalamo-cortical loops, due to increased gain in motor signals, decreased inhibition of those signals, or both [2]. Previous animal and human studies of tic disorders provided support for this theory [3]. Studies of tic disorders have shown hyperactivity in the supplementary motor area and reduced striatal inhibition associated with excessive direct pathway output [2, 4].

However, on a behavioural level, studies of inhibitory control in tic disorders have yielded mixed results. In a meta-analysis of sixty-one studies comprising seven different tests of inhibitory control in 1,717 children and adults with TS, there was a medium effect in favour of inhibitory deficits in TS participants (with comorbid ADHD) compared to healthy controls, and a small yet significant difference between TS participants without ADHD and controls [5]. The presence of comorbid ADHD and the impact of psychiatric medications were identified as possible confounding factors, creating mixed results across studies [5].

Amongst tests of inhibitory control, Morand-Beaulieu et al. [5] found the largest effect sizes in tasks involving verbal responses. In contrast, no significant differences were found between controls and patients with the Tourette syndrome in stimulus-response compatibility tasks or in the Go/No-Go task. The varying results prompt an examination of the different behavioural outcome measures thought to reflect inhibitory control.

Overall, behavioural research supports the idea of inhibitory control as a multidimensional construct [6, 7]. Study findings may therefore depend on which aspects of inhibitory control are affected and whether or not a behavioural task assesses those aspects. However, while variations in task design may have led to varying results, tasks typically used in these studies share features related to prepotent responses. In many oft-used tasks, success may depend on inhibiting a prepotent or overlearned response, through a trial-by-trial sequential presentation of stimuli [5, 6]. Successful inhibitory control in these tasks may entail suppression of a competing response to a stimulus, or suppression of a response to distractors that slows the desired response. These features characterize the Stroop and flanker tasks, sentence completion tasks, circle-tracing task, stimulus-response compatibility tasks, Go/No-Go tasks, and stop-signal tasks.

Aside from variations in task design, disparate results may have arisen from differences in cognitive or attentional demands; and a less demanding task may eliminate differences between patients and controls [5, 8]. Also, most behavioural studies have largely relied on small samples of adult participants, potentially raising concerns about statistical power and developmental inhibitory capacity [5, 8].

Perhaps most importantly, certain tests of inhibitory control may relate more closely to the ability to suppress tics and therefore reveal less about the processes that allow tic expression [6, 7, 9]. It is conceivable that many inhibitory control tasks do not reveal behaviour related to the presumed pathophysiology of tics. As some tasks may correspond to the ability to voluntarily suppress tics, this may explain how patients performed roughly as well as controls. In contrast, some studies may have yielded differences between patients and controls in inhibitory control using a task that is more closely related to the processes that allow tic expression. While an operational definition of inhibitory control can be elusive without measuring brain activity directly, the striatal model of disinhibition may guide selection of a behavioural task and refine its predictions. In this model, tics arise from a cascade of neural overactivity permitted from the basal ganglia, possibly arising from increased signaling to the basal ganglia from cortical areas and by limited inhibitory function within the basal ganglia [3, 9, 10].

The model of striatal disinhibition, in which tics may result from increased gain in motor signals and/or decreased inhibition of those signals, may also encompass deficits in sensory gating, or deficits in filtering out irrelevant stimuli. This could lead to noisier afferent input or greater sensorimotor noise overall [9, 10]. As spontaneous fluctuations in neural activity may generate movement [11], an abnormally increased level of sensorimotor noise could therefore lead to abnormal release of motor signals, manifesting as a failure to suppress internal or external triggers for action [7, 9, 12].

These considerations motivate a novel approach to testing inhibitory control, beyond trial-by-trial tasks requiring inhibition of prepotent responses, instead favouring a dynamic and continuous task entailing simultaneous presentation of multiple stimuli—targets and distractors—as potential targets for action. In a novel task of this nature, successful completion would not require suppression of overlearned responses. Rather, the simultaneous appearance of targets and distractors creates conditions in which participants with tic disorders may behave differently from controls in a manner more consistent with the striatal model, in which noisier sensorimotor processing could lead to abnormal release of motor signals, resulting in the participant acting upon both of these virtual objects. These ideas form the rationale of our current study, in which the performance of children with tic disorders was compared to that of controls in a bimanual object-hit-and-avoid task, in which the participants were presented with moving virtual objects and instructed to strike target objects while avoiding distractor objects. Their behaviour and movement kinematics were captured via the Kinarm, a robotic exoskeleton allowing measurement of kinematic and performance variables. It was hypothesized that participants with tics would hit more distractors than controls, but without striking fewer correct targets and without abnormal movement kinematics in speed and movement area. In contrast to some previous studies, we recruited only children for participation, as the paediatric population is most affected by tic disorders [11, 13].

## 2. Methods

### 2.1. Participants

Sixty-four children diagnosed with the TS or chronic motor tic disorder (mean age 12.38 years; range 7.50-18.42; 93.75% male) were recruited from the Calgary Tourette and Pediatric Movement Disorders Clinic (Calgary, Alberta, Canada) for participation in the study. Inclusion criteria were as follows: diagnosis of the TS or chronic motor tic disorder according to DSM-5 diagnostic criteria; participant and caregiver proficiency in English; and ability to give informed consent. Those with physical limitations preventing interaction with the Kinarm (including height below 126 centimetres) and inability to follow instructions for the study protocol were excluded. Informed consent or assent was obtained for all participants. Study methods were approved by the institutional research ethics board.

Participants' performance in the study task was compared to 146 control children on the same task, drawn from a normative database (mean age 12.99 years; range 6.08-19.92; 52.74% male), through recruitment of volunteers.

### 2.2. Clinical Assessment

Prior to completing the Kinarm task, a clinician administered the Yale Global Tic Severity Scale (YGTSS) to measure current tic severity [14]. Handedness was determined by participant and/or caregiver report. All participants completed diagnostic assessments for the presence of comorbidities including ADHD, obsessive-compulsive disorder (OCD), generalized anxiety disorder (GAD), depression, and autism spectrum disorder (see Table 1) [14–16]. For ADHD, the participants were diagnosed according to diagnostic criteria from the Diagnostic and Statistical Manual of Mental Disorders, Fifth Edition, but did not receive a diagnosis based on screening tests or cutoff scores.

### 2.3. Kinarm

The Kinarm (Kinarm Labs, Kingston, Ontario, Canada) is a robotic exoskeleton that supports the participant's upper limbs against gravity and permits flexion/extension movements of the shoulders and elbows in the horizontal plane. Participants interact with a horizontal virtual reality display. The participant remains in a seated position, adjusted for height using a motorized chair. The Kinarm is fitted to the individual participant and supports the arms, forearms, and hands using troughs. The apparatus records kinematics and streams data to its control software (Dexterit-E version 3.6, Kinarm Labs, Kingston, Ontario, Canada) at 1 kHz.

### 2.4. Object-Hit-and-Avoid Task

**Figure 1:**
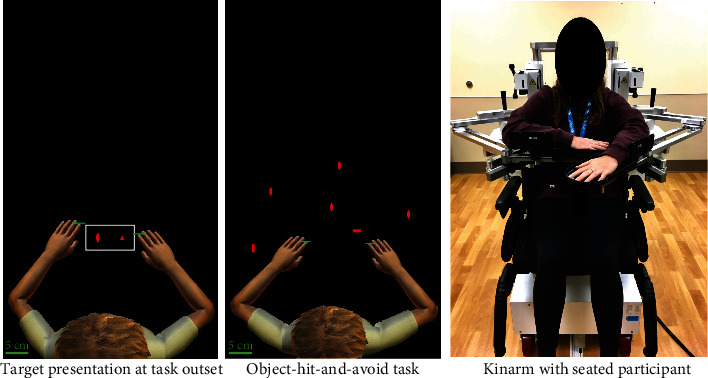
Kinarm and object-hit-and-avoid task.

The object-hit-and-avoid task (Dexterit-E version 3.6, Kinarm Labs, Kingston, Ontario, Canada) was used in the present study (see Figure 1). This bimanual task entails viewing target objects and distractor objects (represented as eight different shapes) moving across the screen simultaneously, with instructions to hit only two target objects while avoiding the six distractor objects, using either hand freely [17]. Five-centimetre paddles represented the hands' positions.

A total of eight shapes were used to represent the objects: square, tall rectangle, short triangle, tall triangle, circle, tall oval, wide rectangle, and wide oval. Two targets were presented for memorization prior to task onset. The participant was instructed to avoid the distractors, represented by the six other shapes. The pair of targets varied for each participant, though in each case, the selected target shapes were of matching width, but of different heights and representing different classes of shapes (i.e., circular versus triangular versus rectangular). The remaining unused shapes comprised the distractors, as well as the two wider shapes. The objects were red in colour, set against a black background. The hand cursors (paddles) were represented as short green horizontal lines.

Objects (targets and distractors) appear pseudorandomly from one of ten locations (bins) 8 centimetres apart, across the top of the screen, and move toward the participant. A total of 300 objects are presented (20 targets and 10 distractors released from each bin) over the course of the task. As the task proceeds, the objects move at faster speeds and appear more often. Objects dropped at an increasing rate according to the following equation:

(1) $$\begin{array}{c} \text{Drop rate}=0.5\text{ objects}/\text{second}+\left[0.025\text{ objects}/\text{second}\times \text{time}\left(\text{s}\right)\right]. \end{array}$$

The speed at which the objects traversed the screen increased according to the following equation:

(2) $$\begin{array}{c} \text{Maximum drop speed}=15\;\text{centimetres}/\text{second}+\left[0.3\text{ centimetres}/{\text{second}}^{2}\times \text{time}\left(\text{s}\right)\right]. \end{array}$$

In this manner, objects initially move at a speed of ~10 cm/s and increase to ~50 cm/s by the end of the task. Task duration is two minutes and eighteen seconds.

### 2.5. Task Parameters

Performance on different aspects of the object-hit-and-avoid task were described using several different variables that have been previously validated [17, 18].

The primary outcome of the study was performance on the variable of *distractor hits*, detailed below.

The *inhibition variables* include distractor hits, distractor proportion, and object processing rate. Distractor hits is defined as the number of distractors hit by the participant, as a percentage of the total number of distractors presented. Distractor proportion refers to the number of distractors hit, as a percentage of the number of total objects hit. Object processing rate is the number of 4targets hit plus distractors missed per second, at 80% of task completion (when performance is at or near maximum).

The *task-level variable* of interest was target hits—a raw count of the number of targets hit correctly (total target hits, as well as target hits using the dominant and nondominant hands).

The *kinematic variables* included hand speed and movement area. Hand speed was measured as the mean hand speed maintained throughout the task, in metres per second. Movement area was defined as the area encompassed by the complete hand path throughout the task, based on a shape that follows the boundaries of the hand's movement trajectory, measured in metres squared. These kinematic variables were measured for the dominant and nondominant hands.

Data pertaining to the task parameters were uploaded from Dexterit-E and extracted for further analysis using MATLAB (R2019a Update 2, Mathworks Inc., MA, USA). Hand speed was filtered using a sixth-order double-pass Butterworth filter with a cutoff filter of 10 Hz.

### 2.6. Statistical Analysis

Independent sample *t* tests were used to search for within-group differences (amongst controls and participants) in the dependent variables between males and females, and between right-handed and left-handed individuals, with the Bonferroni correction. For both participants and controls, the Kolmogorov-Smirnov test was used to test for normality in each dependent variable (see “Task Parameters”).

An analysis of covariance (ANCOVA) was run for each of the task parameters, with age as a covariate, and group (participants with tics but without comorbid ADHD, participants with tics plus comorbid ADHD, and control participants) as the independent variable. For each group, a group mean was calculated for each dependent variable. Analyses included planned contrasts between each of the two participant groups and the control group, as well as post hoc pairwise comparisons between each possible pair of groups (using Bonferroni correction). These statistical tests were performed on the data as they appear in Tables 2–4 (see “Task Parameters”). These analyses were conducted in SPSS (version 23, IBM, Chicago, IL, USA).

As an exploratory analysis, to test for relationships between the dependent variables and tic severity, semipartial correlations were calculated between these variables and the YGTSS total score (controlling for age), to search for unique relations between each of these variables and YGTSS total score, thereby excluding variance explained by the other variables. Semipartial correlations were run within each category of dependent variable (e.g., inhibition variables, task-level variables, and kinematic variables).

Age-predicted norms were calculated for each task parameter (i.e., for each dependent variable). A second-order polynomial curve was fitted to the control participants' performance (across the range of their ages) in each dependent variable, including calculation of 95% prediction interval bands. Therefore, for each participant, impairments could be detected that fell outside age-predicted norms.

## 3. Results

### 3.1. Participants

Demographic characteristics were similar across groups (see Table 1). No statistically significant differences were found between males and females, or between left-handed and right-handed individuals, in any task parameter, within the controls or within the participants. For participants and controls, data pertaining to each task parameter were not normally distributed (as per the Kolmogorov-Smirnov test for each task parameter, *p* < 0.001).

### 3.2. Task Parameters

Participants with tics (both with and without comorbid ADHD) performed below the level of the control group in the *inhibition variables* making significantly more distractor hits, scoring a higher distractor proportion, and scoring a lower object processing rate (Table 2). Post hoc pairwise comparisons between participants with tics with and without comorbid ADHD showed no statistically significant differences in distractor hits, distractor proportion, or object processing rate.

Participants with comorbid ADHD struck fewer targets than controls. When examining targets hit by the dominant or the nondominant hand, the number of targets hit by the nondominant hand was lower in participants with comorbid ADHD than in controls. However, the number of targets hit by participants without comorbid ADHD (whether examining total target hits or target hits by either hand) failed to differ significantly from controls (Table 3).

**Figure 2:**
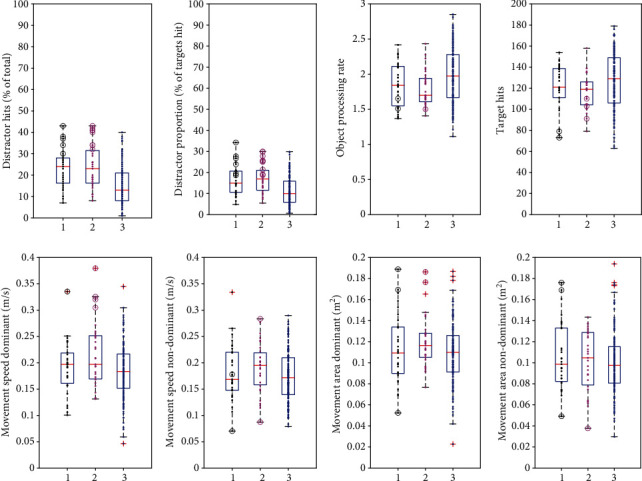
Box-and-whisker plots comparing patients without comorbid ADHD with those with comorbid ADHD and controls. In each plot, group 1 denotes patients without comorbid ADHD, group 2 denotes patients with comorbid ADHD, and group 3 denotes controls. Circles represent individuals scoring outside age-predicted norms (black denotes patients without comorbid ADHD; magenta denotes patients with comorbid ADHD). Crosses represent outliers (more than 1.5 times the interquartile range away from the top or bottom of the box). Dots represent each individual participant.

In the kinematic variables, significant differences from controls were found only in participants with comorbid ADHD. These participants showed significantly greater movement speed than controls in both the dominant and nondominant hands (see Table 4). Participants with comorbid ADHD also covered a larger movement area using the dominant hand than controls, though the difference between this participant group and controls in movement area covered by the nondominant hand failed to reach statistical significance. Participants without comorbid ADHD did not differ significantly from controls in movement speed or movement area, for either the dominant or nondominant hand (Table 4; Figure 2).

### 3.3. Performance Relative to Age-Predicted Norms

**Figure 3:**
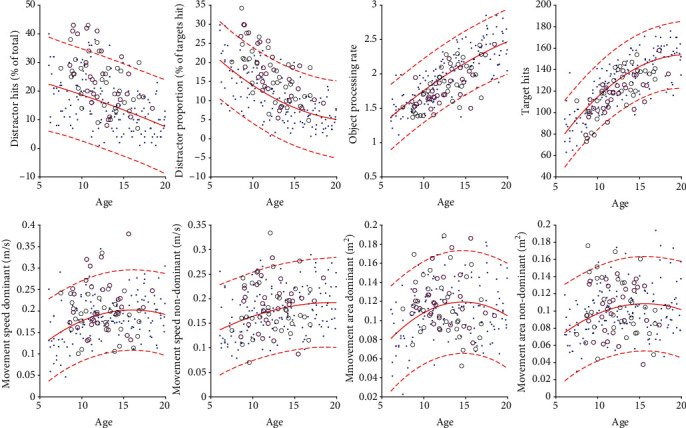
Performance on different task parameters as a function of age, comparing patients (those without comorbid ADHD with those with comorbid ADHD) with controls. In each plot, dots represent controls, black circles represent patients without comorbid ADHD, and magenta circles represent patients with comorbid ADHD. Age curves are shown as solid traces, with 95% prediction bands shown as dotted traces.

**Figure 4:**
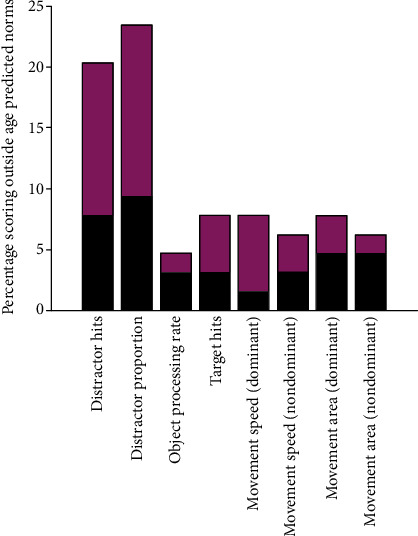
Percentage of patients who scored outside age-predicted norms in the task parameters. Each bar is divided to show patients without comorbid ADHD (in black) and those with comorbid AHDH (in magenta).

In addition to group mean differences described above, individual behaviour was examined by determining how many participants fell outside age-predicted norms. There was a significant effect of age on performance in all task parameters (Table 2).  Figure 3 shows age curves for participants and controls, with 95% prediction bands superimposed, showing individual participants who scored outside age-predicted norms. The performance of individual participants scoring outside age-predicted norms is also shown alongside group data in a box-and-whisker plot (Figure 2). The parameters for which a notably large percentage performed abnormally were the inhibition variables of distractor proportion (15/64; 23.44%) and distractor hits (13/64; 20.31%). In the remaining task parameters, fewer than 10% fell outside age-predicted norms (Figure 4).

### 3.4. Correlations with Tic Severity

A weak negative semipartial correlation was found between the YGTSS total score and distractors hit with the nondominant hand (sr -0.27; sr^2^ 0.08; *p* = 0.03). No other dependent variables explained a statistically significant unique portion of variance in the YGTSS total score, adjusting for age.

## 4. Discussion

The principal findings from our study were that participants with tic disorders hit a greater number of distractors than controls. The group with tic disorders (without comorbid ADHD) hit a greater number of distractors than controls, though without a decrease in performance in terms of correctly striking targets. Importantly, in this group, the tendency to hit distractors occurred without statistically significant changes in the kinematic variables of movement speed and movement area. In contrast, participants with comorbid ADHD differed from controls not only by striking more distractors but also by striking fewer targets. Those with comorbid ADHD also differed from controls in motor behaviour in that they moved at a greater average speed and covered a larger area using the dominant hand. When looking at performance on an individual rather than group level, performance was abnormal for a large minority of participants, with over 20% performing outside the age-predicted norm, whereas fewer than 10% did so in the other task parameters.

Separately analyzing performance of participants with and without comorbid ADHD reduced the confounding effect of inattentiveness/hyperactivity on performance. Conceivably, attention deficits may have accounted for reduced allocation of attention to the nondominant side of the workspace [19]. Greater average movement speed and movement area may also have been a result of inattentiveness/hyperactivity, related to ADHD-specific deficits in upper limb function [20], hyperactive behaviour, and/or compensatory increases in speed from delayed reaction time.

The findings are consistent with previous studies showing impaired inhibitory control in tic disorders, insofar as participants with tics demonstrated an increased tendency to erroneously perform the incorrect action by striking distractors. This is in line with other behavioural tasks in which participants with tics responded incorrectly to stimuli by performing an undesired, nontarget action [5, 21]. Expanding on the existing literature, the tendency of participants without comorbid ADHD to strike distractors did not come at the cost of lower task performance (i.e., striking correct targets).

Many studies of inhibitory control to date have relied on tasks in which participants' successful performance is based on the ability to suppress a prepotent or overlearned response [5, 6], requiring the participant to suppress a competing response to a stimulus, or to suppress a response to distractors that slows the desired response. However, these behavioural tasks may not be closely related to the presumed pathophysiology of tics. In this regard, an advantage of the object-hit-and-avoid task is its simultaneous presentation of multiple, mobile stimuli as potential targets for action. As a result, this task may be better suited to testing the idea that inhibitory control deficits associated with tic disorders are related to increased sensorimotor noise. This may reflect abnormal sensory gating in which irrelevant stimuli fail to be ignored, or increased sensorimotor noise overall allowing an abnormal release of motor signals. In the human motor system, under normal circumstances, a gradual buildup of neuronal activity known as “readiness potential” precedes voluntary movement, but when there is a weak imperative to generate movement, the moment at which the threshold is crossed is determined by spontaneous fluctuations in neural activity below this threshold [11]. An abnormally increased level of sensorimotor noise, generating suprathreshold fluctuations, could therefore lead to abnormal release of motor signals [11]. Importantly, this does not necessarily predict that erroneous performance of an incorrect action disrupts performance of the correct target action.

In participants without comorbid ADHD, for whom the potentially confounding effect of inattentiveness is mitigated, our findings support the prediction that participants with tics would strike more distractors than controls but not at the cost of striking fewer correct targets. The findings therefore adhere to the notion that participants with tics act upon both targets and distractors, in accordance with a reduced ability to suppress a response to potential triggers for action. This may be related to increased sensorimotor noise or abnormal sensory gating, though other explanatory frameworks for tics would arguably predict similar findings.

One controversy surrounding tics and their relationship with disordered inhibitory control is whether or not these can be explained by stimulus-driven actions. It has been argued that the human motor system is continually presented with potential triggers for action and that under normal circumstances, actions driven by these affordances are inhibited [7, 9, 17]. This is a particularly controversial conceptual framework for tics, as they rarely resemble stimulus-driven actions [9], though an affordance-like mechanism may be at play in the generation of tics, in that an inhibitory control deficit could also fail to suppress internal triggers for action [7]. The object-hit-and-avoid task presents several objects that can be acted upon and may therefore test the ability to resist potential triggers for action. The participants' performance in the current study may thus be consistent with an abnormal affordance-like mechanism, given their tendency to hit distractors but without failing to also hit targets, in the case of participants without comorbid ADHD.

The current study has limitations. The object-hit-and-avoid task differs from commonly used behavioural tasks in the study of inhibitory control, limiting direct comparisons to previous work. The object-hit-and-avoid task was not explicitly designed to distinguish between different components of inhibitory control, such as cognitive (explicit) or subliminal (implicit) processes [8, 22, 23], and so, it is not clear which components were tested. Overall, how closely performance on any given behavioural task relates to the presumed pathophysiology of tics is subject to debate.

Another potential limitation is the skewed sex ratio of the study sample. However, there were no statistically significant differences between males and females in any task parameters, within the controls or within the patients. Moreover, studies show that tic disorders are more common in boys, by a ratio of 4 or 5 to 1 [16]. It must be acknowledged that medications were not stopped for participation in the study, and therefore, this may have affected performance on the task [24, 25].

Results were not analyzed based on the presence or absence of comorbidities outside ADHD. ADHD is the most frequent comorbidity in TS [26], and we hypothesized that it would have the most important impact on performance. There is evidence supporting upper-limb motor control abnormalities in those with ADHD [20], executive function deficits that include reduced response inhibition [3], and diminished cortical inhibition in ADHD comorbid with the Tourette syndrome [27]. Though comorbid ADHD was recognized as an important phenotypic subtype, another limitation of the present study is that a “pure” ADHD group (without tics) was not recruited. It must also be acknowledged that control participants were included on the basis of self-reported absence of psychiatric conditions, as opposed to formal scores using clinimetric scales. Relatedly, the data were not analyzed according to severity of ADHD.

In the striatal disinhibition model, tic disorders are associated with increased sensorimotor noise, conveyed by excessive cortical signaling to the basal ganglia and by limited inhibitory function within the basal ganglia; and this cascade of neural overactivity may underlie tics, as well as a diminished capacity to filter out irrelevant stimuli. This model predicts a more indiscriminate striking of targets and distractors by patients with tics versus controls. This model could also predict supranormal performance by patients with tics. Some previous studies using different behavioural tasks have found evidence of superior performance [5], though it is possible that these tasks corresponded to the ability to voluntarily suppress tics, explaining how patients performed at least as well as controls or better. In contrast, the object-hit-and-avoid task likely serves as a better behavioural proxy for the striatal disinhibition model of tics, though it may simply be indicative of the biological trait of having tics, rather than a direct demonstration of the mechanisms that give rise to tics. The novelty of the task is therefore a strength of this study. Additional strengths of this study include the use of a large, well clinically phenotyped group of children with tic disorders.

As it represents a new approach to the study of inhibitory control in tic disorders, the clinical relevance of hitting distractors in the object-hit-and-avoid task remains to be demonstrated. The only correlation with tic severity found in the current study was a negative correlation with distractors hit by the nondominant hand. As previous studies have suggested that suppression of tics is associated with a reduction in the normal lateralization of motor function [28], it is possible that participants with lower tic severity in the current study relaxed inhibition of the nondominant side [29]. This reduced lateralization is perhaps an epiphenomenon of tic suppression [28]. However, measuring correlations between task performance and tic severity in our study was exploratory in nature. As noted above, the tendency to strike distractors may simply be part of the biological trait of tic disorders and unrelated to tic severity. This is in line with previous studies showing that impaired automatic inhibition [7] and excessive grip force during object manipulation [30] are not correlated with tic severity. A meta-analysis by Morand-Beaulieu et al. found that inhibitory deficits were associated with the YGTSS total tic severity score [5].

In summary, the current study represents a novel approach to the study of inhibitory control in children with tic disorders, yielding further evidence of deficient inhibitory control. Our study motivates future research in this area, including longitudinal study of participants with tic disorders and the evolution of their performance alongside treatments. Further study is needed to explore the relationship between Kinarm performance deficits and clinically relevant outcomes.

## Figures and Tables

**Table 1:** Participant demographic variables.

	Controls (*n* = 146)	Tic disorders group (*n* = 64)	Tics without comorbid ADHD (*n* = 33)	Tics with comorbid ADHD (*n* = 31)
Mean age (standard deviation)	12.99 (4.10)	12.38 (2.58)	12.53 (2.70)	12.38 (2.51)
Left-hand dominant (percent of total)	13/146 (8.9%)	11/64 (17.19%)	3/33 (9.09%)	8/31 (25.81%)
Male : female ratio (percent male of total)	77 : 69 (52.74%)	60 : 4 (93.75%)	28 : 5 (84.85%)	30 : 1 (96.77%)

Comorbidities in tic disorders group				
Attention-deficit hyperactivity disorder		31/64 (48%)		
Obsessive-compulsive disorder		16/64 (25%)	6/33 (18.18%)	10/31 (32.26%)
Generalized anxiety disorder		16/64 (25%)	8/33 (24.24%)	8/31 (25.81%)
Depression		5/64 (7.8%)	3/33 (9.09%)	2/31 (6.45%)
Autism spectrum disorder		5/64 (7.8%)	1/33 (3.03%)	4/31 (12.9%)

Medications for tics in tic disorders group (number of participants and percentage of group)				
Aripiprazole	5 (7.81%)			
Clonidine	4 (6.25%)			
Guanfacine	4 (6.25%)			
Risperidone	1 (1.56%)			
Topiramate	1 (1.56%)			
No medications for tics	49 (76.56%)			

**Table 2:** ANCOVA results for each inhibition variable, control participants versus those with tic disorders, with age as covariate; and for control participants versus those with tic disorders, groups are split into two (those without ADHD and those with ADHD), with age as covariate, and planned contrasts comparing each of the two patient groups to the control group.

Inhibition variables	Controls (adjusted mean and 95% CI)	Tics-without-ADHD	*p* value versus controls	Tics-plus-ADHD	*p* value versus controls	Between-groups *F*-stat and *p* value	Post hoc comparisons between patient groups (mean difference and 95% CI for difference)
Distractor hits	15.61% (14.28, 16.95)	22.71% (19.81, 25.61)	<0.0001	23.56% (20.66, 26.46)	<0.0001	*F* = 18.513; *p* < 0.0001	-0.085; -5.87, 4.17; *p* = 0.99
Distractor proportion	11.31% (10.48, 12.15)	15.99% (14.17, 17.81)	<0.0001	16.49% (14.68, 18.31)	<0.0001	*F* = 20.339; *p* < 0.0001	-0.50; -3.65, 2.64; *p* = 0.99
Object processing rate	1.97 (1.93, 2.00)	1.87 (1.79, 1.95)	0.032	1.83 (1.75, 1.91)	0.002	*F* = 6.202; *p* = 0.002	-0.05; -0.18, 0.10; *p* = 0.99

**Table 3:** ANCOVA results for each task-level variable, control participants versus those with tic disorders, with age as covariate; and for control participants versus those with tic disorders, groups are split into two (those without ADHD and those with ADHD), with age as covariate, and planned contrasts comparing each of the two patient groups to the control group.

Task-level variables	Controls (adjusted mean and 95% CI)	Tics-without-ADHD	*p* value versus controls	Tics-plus-ADHD	*p* value versus controls	Between-groups *F*-stat and *p* value	Post hoc comparisons between patient groups (mean difference and 95% CI for difference)
Target hits	126.86 (124.35, 129.36)	122.66 (117.20, 128.13)	0.157	119.74 (114.28, 125.21)	0.018	*F* = 3.341; *p* = 0.037	2.92; -6.536, 12.732; *p* = 0.99
Target hits dominant	65.87 (60.59, 65.54)	63.73 (60.15, 67.31)	0.268	62.91 (59.33, 66.48)	0.130	*F* = 1.522; *p* = 0.221	0.82; -5.36, 7.011; *p* = 0.99
Target hits nondominant	60.99 (59.37, 62.60)	58.93 (55.41, 62.46)	0.283	56.84 (53.31, 60.34)	0.034	*F* = 2.540; *p* = 0.081	2.10; -4.01, 8.20; *p* = 0.99

**Table 4:** ANCOVA results for each kinematic variable, control participants versus those with tic disorders, with age as covariate; and for control participants versus those with tic disorders, groups are split into two (those without ADHD and those with ADHD), with age as covariate, and planned contrasts comparing each of the two patient groups to the control group.

Kinematic variables	Controls (adjusted mean and 95% CI)	Tics-without-ADHD	*p* value versus controls	Tics-plus-ADHD	*p* value versus controls	Between-groups *F*-stat and *p* value	Post hoc comparisons between patient groups (mean difference and 95% CI for difference)
Movement speed dominant	0.183 m/s (0.175, 0.192)	0.193 m/s (0.175, 0.211)	0.342	0.216 m/s (0.198, 0.234)	0.001	*F* = 5.317; *p* = 0.006	-0.023; -0.054, 0.010; *p* = 0.23
Movement speed nondominant	0.173 m/s (0.165, 0.181)	0.182 m/s (0.166, 0.199)	0.326	0.192 m/s (0.175, 0.209)	0.047	*F* = 2.204; *p* = 0.113	-0.010; -0.038, 0.019; *p* = 0.99
Movement area dominant	0.109 m^2^ (0.104, 0.114)	0.113 m^2^ (0.103, 0.123)	0.497	0.120 m^2^ (0.110, 0.131)	0.047	*F* = 2.052; *p* = 0.131	-0.010; -0.025, 0.010; *p* = .91
Movement area nondominant	0.100 m^2^ (0.095, 0.104)	0.106 m^2^ (0.096, 0.117)	0.235	0.102 m^2^ (0.092, 0.112)	0.650	*F* = 0.740; *p* = 0.478	0.004; -0.014, 0.022; *p* = 0.99

## Data Availability

The authors of this work have agreed to make the study data available upon request, directly through one of the authors, via the contact information: Tamara Pringsheim, 3280 Hospital Drive NW, Calgary AB T2N 4Z6, tmprings@ucalgary.ca

## Abbreviations

ADHD: Attention-deficit hyperactivity disorder
ANCOVA: Analysis of covariance
DSM-5: Diagnostic and Statistical Manual of Mental Disorders, 5^th^ Edition
GAD: Generalized anxiety disorder
OCD: Obsessive-compulsive disorder
TS: Tourette syndrome
YGTSS: Yale Global Tic Severity Scale.

## Additional Points

*Key Points*. Tics may result from sensorimotor disinhibition. Different behavioural tasks have been used to study inhibitory control in patients with tics, yielding mixed results. Many tasks may be unrelated to the presumed pathophysiology of tics (e.g., striatal disinhibition and predicting deficits in filtering out irrelevant stimuli). Therefore, our novel approach entailed simultaneous presentation of multiple stimuli—targets and distractors—as potential targets for action. Accounting for comorbid ADHD addressed confounding inattentiveness/hyperactivity. Those with tics (without comorbid ADHD) hit more distractors than controls, though not at the cost of correctly striking targets, and without changes in movement speed/area. Those with comorbid ADHD differed from controls not only by striking more distractors but also by striking fewer targets.
